# PCB-YOLO: Enhancing PCB surface defect detection with coordinate attention and multi-scale feature fusion

**DOI:** 10.1371/journal.pone.0323684

**Published:** 2025-06-02

**Authors:** Ze Wei, Fan Yang, Kezhen Zhong, Linkun Yao

**Affiliations:** 1 School of Electronic and Information Engineering, Hebei University of Technology, Tianjin, China; 2 Faculty of Computing, Harbin Institute of Technology, Harbin, China; Universite Cote d'Azur, FRANCE

## Abstract

Nowadays, industrial electronic products are integrated into all aspects of life, with PCB quality playing a decisive role in their performance. Ensuring PCB factory quality is thus crucial. Common PCB defects serve as key references for evaluating quality. To address low detection accuracy and the bulky size of existing models, we propose an improved PCB-YOLO model based on YOLOv8n.To reduce model size, we introduce a novel CRSCC module combining SCConv convolution and C2f, enhancing PCB defect detection accuracy and significantly reducing model parameters. For feature fusion, we propose the FFCA attention module, designed to handle PCB surface defect characteristics by fusing multi-scale local features. This improves spatial dependency capture, detail attention, feature resolution, and detection accuracy. Additionally, the WIPIoU loss function is developed to calculate IoU using auxiliary boundaries and address low-quality data, improving small-target recognition and accelerating convergence. Experimental results demonstrate significant improvements in PCB defect detection, with mAP50 increasing by 5.7%, and reductions of 13.3% and 14.8% in model parameters and computational complexity, respectively. Compared to mainstream models, PCB-YOLO achieves the best overall performance. The model’s effectiveness and generalization are further validated on the NEU-DET steel surface defect dataset, achieving excellent results. The PCB-YOLO model offers a practical, efficient solution for PCB and steel defect detection, with broad application prospects.

## Introduction

As the basic component of the electronic industry, printed circuit board (PCB) is widely used in all kinds of electronic equipment in life and production [1–4]. The quality of PCB directly affects the performance and life of electronic equipment. Therefore, defect detection of PCB is a key and necessary task in electronic manufacturing industry. Flying pin testing is an electrical testing method that is mainly used to detect short and open circuit faults on circuit boards. This method utilizes multiple fast-moving electrical probes driven by motors to contact the pins of the circuit board, thereby making electrical measurements, instead of a conventional needle bed [5]. As a traditional PCB inspection method, manual inspection relies on the visual inspection of production line operators, and usually requires the help of magnifying glass to identify defects on complex circuit boards. As the scale of integrated circuits increases, so does the complexity of printed circuit boards, which leads to increased difficulty in contact measurement and manual inspection. Traditional manual detection methods are costly and inefficient, and are gradually being eliminated. Using efficient automated defect detection algorithms can not only significantly improve production efficiency, but also greatly reduce labor costs. In short, the application of automated defect detection technology not only optimizes the production process, but also promotes the rational allocation of human resources.

Before deep learning technology was widely used, PCB defect detection mainly relied on traditional image processing and machine learning methods. These traditional methods face some challenges, such as sensitivity to data distribution, insufficient generalization ability, and inefficient detection. Wang et al. [6] proposed an on-line PCB defect detection technology based on machine vision, which firstly preprocesses the image, including operations such as smoothing, contrast enhancement and sharpening, to improve the image quality; Then, the image is processed and binarized, and the hybrid method of mathematical morphology and pattern recognition is used for defect recognition and detection; Finally, the reference image obtained by mathematical morphology method is used as the system self-inspection template, and the image difference detection algorithm is introduced to segment the defect image, remove redundant points and mark the recognition results. Yuk et al. [7] implemented PCB defect detection using accelerated robust features and random forest algorithms. By considering the density of features, a weighted kernel density estimation mAP is generated with weighted probability, and the detection of defect concentration area is realized. Some scholars have also proposed PCB surface defect detection methods based on machine learning, but this method is not a real-time method [8,9]. Although machine learning methods can identify defects on PCB surfaces, many algorithms still rely on manual setting of image features, which usually needs to be based on prior knowledge. This dependency limits the generalization ability of the algorithm.

With the development of deep learning technology [10–16], using deep learning technology to solve PCB defect detection has gradually become the mainstream research method [17–19]. Huang et al. [20] improved the YOLOv5s architecture by introducing deeply separable convolution and spatial attention mechanisms, using an adaptive spatial feature fusion module, and proposed an SSA-YOLO model, which effectively solved the conflict information between features in different layers, enabling the model to better adapt to the detection requirements of complex scenes and multi-scale targets. Zhang et al. [21] developed a lightweight algorithm YOLO-RRL based on YOLOv8 architecture for the problems of various types of PCB surface defect detection and large amount of model calculation, and used RFD method to optimize the downsampling process of shallow and deep feature mAPs; RepGFPN is used to optimize the feature fusion network to improve the fusion efficiency of feature mAPs with different scales; Finally, a lightweight asymmetric detection head is used to reduce the amount of model parameters and calculation while maintaining the detection accuracy. References [22] and [23] apply YOLOv7 model and YOLOv5s model to PCB defect detection respectively, and achieve fast and accurate detection effects by adding convolution layer and upsampling layer, introducing attention mechanism and replacing activation function. In order to better detect small defects in PCB, Xu et al. [24] added a detection layer and anchor frame dedicated to detecting small targets to the YOLOv5s model. Inspired by the YOLOX model, a novel decoupling head mechanism was designed to decouple the three tasks of classification prediction, position prediction and confidence prediction, which accelerated the convergence speed of the model. The improved model has good adaptability and robustness. Xiao et al. [25] improved the lightweight YOLOv7-tiny model by introducing coordinate attention mechanism, deep separable convolution and Inner-CIoU loss function, and constructed an efficient PCB defect detection model CDI-YOLO, which balanced the contradiction between accuracy, speed and parameter quantity and achieved accurate defect detection. Jiang et al. [26] designed the DCR-YOLO model by drawing lessons from the overall structure of YOLO series models and the idea of target detection. The model uses a backbone network composed of double cross residual blocks and ordinary residual blocks, and proposes a PCR module to enhance the feature fusion mechanism between feature layers of different scales. The SSDT-FPN module and C5ECA module are designed to improve the feature fusion layer and self-made feature weights respectively, and the average detection accuracy is 98.58%. To sum up, the application of deep learning technology has significantly improved the efficiency and accuracy of PCB defect detection. Different research teams have proposed a variety of effective solutions for the particularity of PCB defects by improving and optimizing the existing network architecture.

In recent years, although deep learning-based PCB defect detection methods have made significant progress, they still face some challenges in practical applications. Existing methods often perform poorly when dealing with tiny defects in complex backgrounds, resulting in high missed detection rates. At the same time, many models have deficiencies in feature extraction and context understanding, limiting their generalization capabilities in diverse PCB scenarios. In addition, the accuracy of bounding box regression is also a common problem in existing methods, which directly affects the precise localization of defects. In response to these key issues, we propose an improved PCB-YOLO model, which aims to fill the shortcomings of existing research. Our model is based on YOLOv8, an efficient and smaller parameter object detection algorithm suitable for the deployment of industrial inspection equipment. We improve YOLOv8 in the following three aspects:

(1) To solve the problem of possible loss of feature information in lightweight design, we introduce the CRSCC module, which enhances the model’s capabilities in feature extraction and context awareness, enabling the model to better understand complex scenes, thus improving the generalization ability of detection.
(2) In view of the characteristics that PCB defects are usually small in size and have complex backgrounds, we design the FFCA module, which is a coordinate attention mechanism module that fuses multi-scale local features. By combining local and global features and introducing coordinate information, it effectively improves the model’s ability to capture the details of PCB surface defects, thereby improving the accuracy of detection.
(3) To improve the regression accuracy of the bounding box, we propose the WIPIoU loss function, which is the advantage of combining the three loss functions of Wise-IoU, MPDIoU, and Inner-IoU. The WIPIoU loss function not only enables the model to locate the target more accurately, but also accelerates the convergence rate of the model.

Through these improvements, our PCB-YOLO model can handle tiny defects in complex backgrounds more effectively, improving the accuracy and efficiency of inspection, thus bringing a more efficient and accurate solution to the field of PCB defect inspection.

## Materials and methods

### Dataset

**Fig 1:**
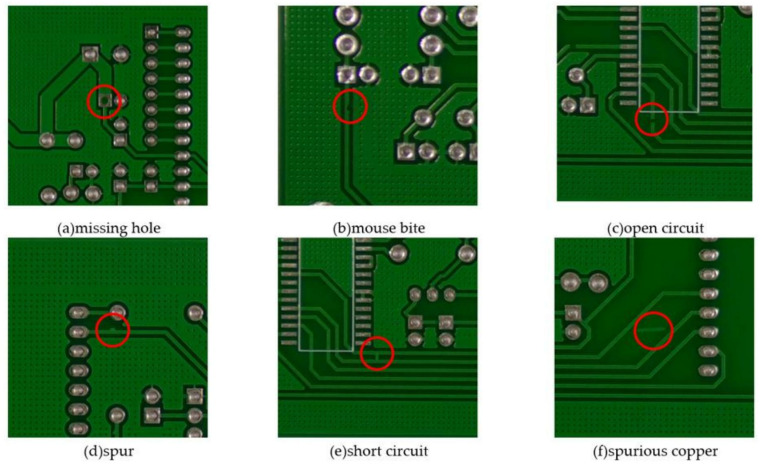
Images of different PCB surface defect types.

**Fig 2:**
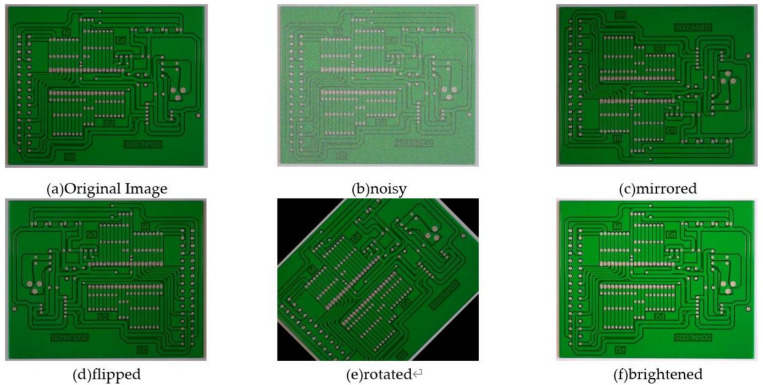
Data augmentation example diagram.

In this paper, the open source PCB defect dataset PKU-Market-PCB [27] of Peking University Intelligent Robot Open Laboratory is used, with a total of 673 PCB defect images, some of which are shown in Fig 1. This data set involves a total of 6 types of defects, including missing hole, mouse bite, open circuit, short circuit, spur, and spurious copper, including 115 missing holes, 115 mouse bites, 116 open circuits, 116 short circuits, 115 burrs, 116 fake copper. Due to the limited number of various PCB defect samples in this public data set, and the samples collected in an ideal environment, it cannot meet the requirements of model training. Therefore, first, the data of the existing samples is enhanced, and the data set is expanded to 4038 through operations such as rotation, mirroring, noise and brightness adjustment. Some of the expanded sample images are shown in Fig 2. During the experiment, the data set is randomly divided into training set, verification set and test set according to the ratio of 7:2:1, that is, 2825 images are used as the training set of the training model, 808 images are used as the verification set, and 405 images are used as the test set. The final number of each defect type is shown in the Table 1.

**Table 1:** PKU-market-PCB dataset defect description and quantity.

Defect Type	Defect description	Number of Original Images	Number of Images After Expansion
missing hole	Failure to drill some holes correctly	115	690
mouse bite	Circuit board breakage	115	690
open circuit	Solder joint interruption	116	696
spur	Excess metal fragments appear	115	690
short circuit	Undesirable current path between solder joints	116	696
spurious copper	Copper foil layer fails to cover correctly	116	696
Total number	/	673	4038

### PCB-YOLO model

In this study, we chose the YOLOv8 model as our benchmark model, and this choice is based on multiple considerations. First, the YOLOv8 model has made significant improvements in architecture and training methods based on previous YOLO versions. Improved model structure design, enhanced feature extraction techniques, and optimized training methods are integrated. What really makes the YOLOv8 model stand out is its impressive combination of speed, accuracy, and efficiency, making it one of the most powerful models ever made by Ultralytics. With an improved design, YOLOv8 provides better feature extraction, the process of identifying important patterns and details from images, capturing complex aspects more accurately even in challenging scenes. However, the YOLOv8 model has some shortcomings in practical application. For example, the model has a large amount of parameters, which makes it difficult to deploy on resource-constrained platforms such as mobile devices.

This challenge is particularly important for our research, because in practical application scenarios, PCB defect detection involves real-time processing of multiple frames of images, so in large-scale defect detection tasks, detection speed is crucial. At the same time, YOLOv8 model. There is still room for improvement in the detection accuracy. Although the YOLOv8 model performs well in some aspects, it needs further optimization and adjustment, because our goal is to develop an efficient and practical target detection system.

**Fig 3:**
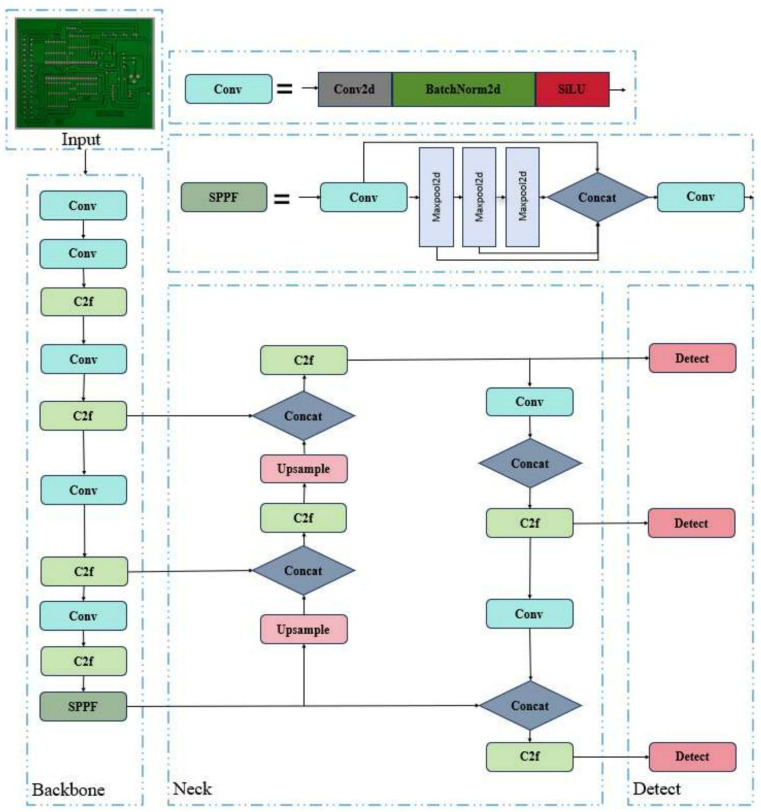
YOLOv8 model structure.

The backbone part of YOLOv8 is basically the same as that of YOLOv5. Based on the CSP idea, the C3 module is replaced with the C2f module. The C2f module draws lessons from the ELAN idea in YOLOv7 [28], and combines C3 and ELAN to form the C2f module, so that YOLOv8 can obtain richer gradient flow information while ensuring lightweight. At the end of the backbone network, the most popular SPPF module is still used, which ensures the detection accuracy of objects at different scales. In the neck part, the feature fusion method used by YOLOv8 is PAN-FPN, which strengthens the fusion and utilization of feature layer information at different scales. The head network generates a detection box by applying the anchor box to the multi-scale feature mAP of the neck network, which shows the category, location and confidence of the target. The structure of the YOLOv8 model is shown in Fig 3.

According to different network depth and width, YOLOv8 can be divided into 5 versions: YOLOv8n, YOLOv8s, YOLOv8m, YOLOv8l, YOLOv8x. Five versions of the YOLOv8 model were used to train the PCB surface defect data set. The training results are shown in Table 2. The YOLOv8n model has the smallest amount of parameters and calculation, and the average accuracy is also high. It is suitable for running on devices with limited computing resources and is very suitable for embedded systems or mobile devices. Based on the consideration of lightweight and the need for accuracy, this paper selects YOLOv8n as the benchmark model.

**Table 2:** Training results of different sizes of YOLOv8 on PKU-market-PCB dataset.

Model	Parameters(MB)	FLOPs (G)	Precision/%	Recall/%	mAP50/%
YOLOv8n	3.0	8.1	85.1	84.6	90.4
YOLOv8s	11.1	28.4	95.4	76.3	91.2
YOLOv8m	25.8	78.7	94.4	89.9	95.5
YOLOv8l	43.6	164.8	90.5	90.1	95.0
YOLOv8x	68.1	257.4	90.1	90.3	95.3

#### Model lightweight improvement.

In the field of PCB defect detection, the identification and location of small targets has always been a challenging task. Since defects are usually small in size and difficult to distinguish in complex PCB backgrounds, traditional feature extraction backbone networks based on convolutional neural networks (CNNs) often face challenges. Since small targets occupy a small proportion of pixels in the original image, they are easily submerged in a large amount of redundant information during the downsampling process, resulting in degraded detection performance. Although the structure of C2f module in the backbone network of YOLOv8n model is relatively simple and has good feature fusion ability, with the complexity of application scenarios, we realize that C2f module has certain limitations in processing context information. Although the traditional Conv1 and Conv2 layers in C2f can extract features, they often ignore the spatial context relationship between features, which is especially obvious when dealing with images with complex backgrounds. SSconv convolution allows the network to not only consider local information when processing images, but also combine global contextual information, which helps improve the model’s ability to understand complex scenes and improve the final prediction results.

Therefore, we propose a brand-new CRSCC module based on SCConv convolution [29] and C2f module to reduce the redundant information of the feature mAP in both space and channel aspects, enhance the model’s understanding of the spatial context, and thus improve the quality of feature extraction.

**Fig 4:**
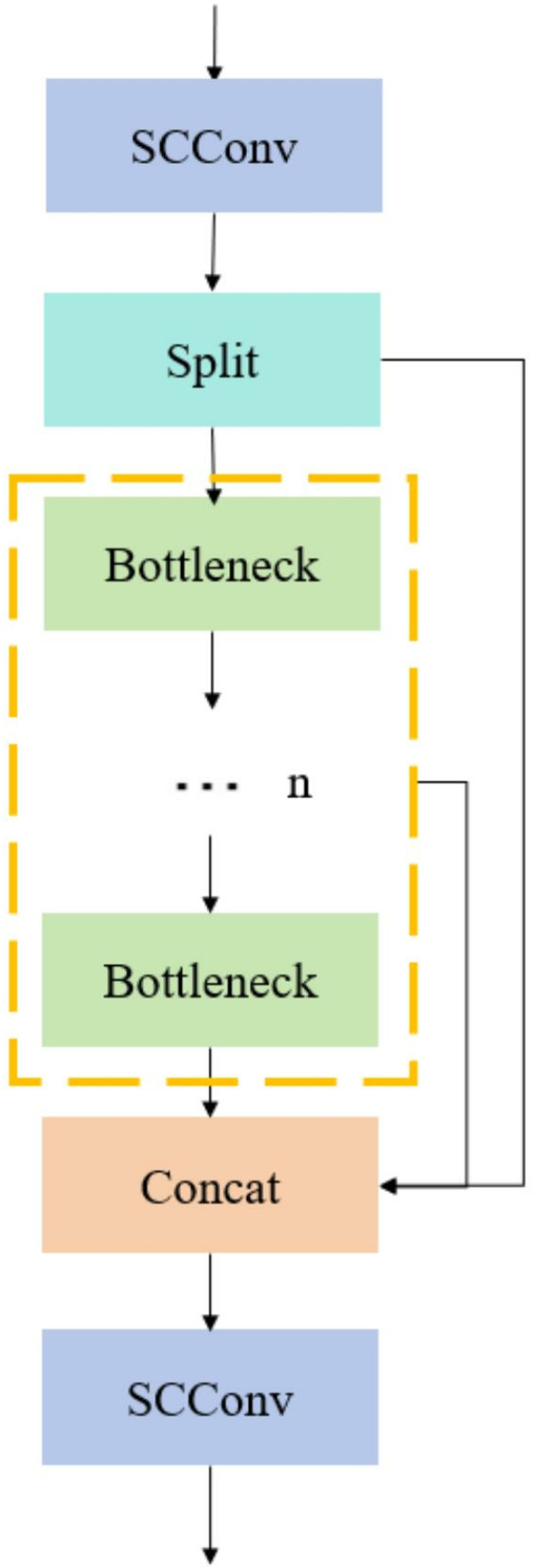
CRSCC structure diagram.

**Fig 5:**
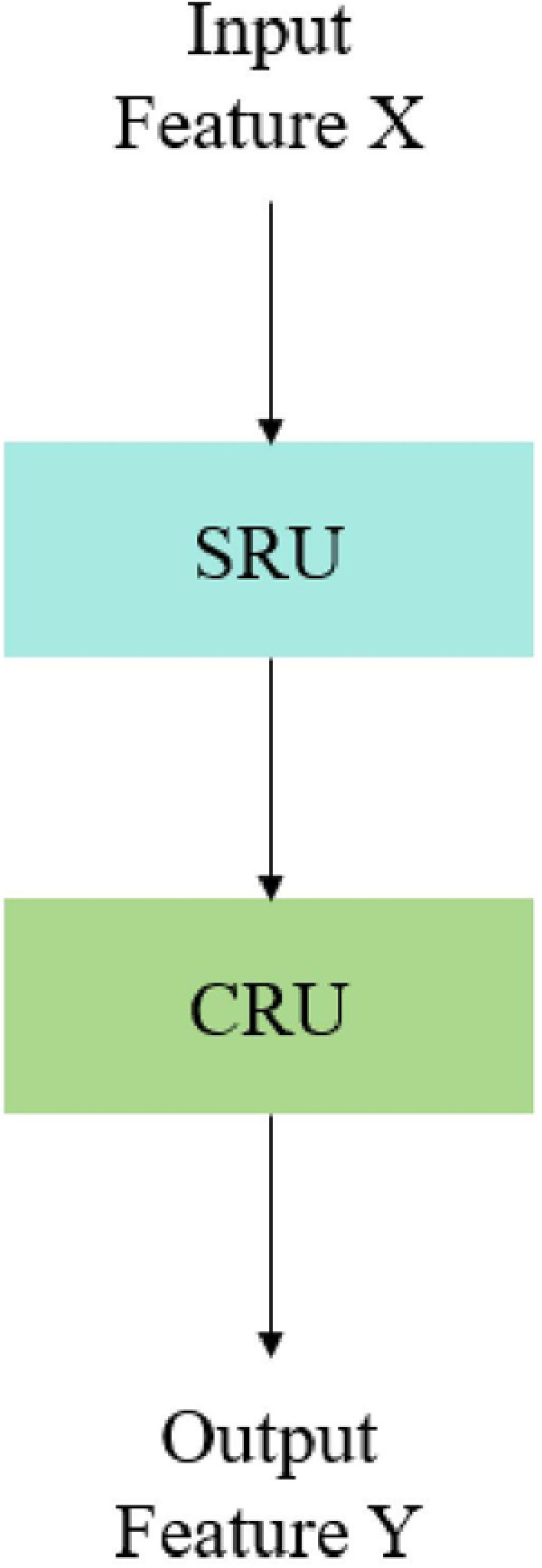
SCConv structure diagram.

In the CRSCC module shown in Fig 4, we have taken several improvements to enhance network performance. First, we optimize the original C2f module and replace two standard convolution operations with the SCConv module. The purpose of this replacement is to strengthen the ability of the backbone network in spatial context modeling, so that the network can understand the spatial relationship between features more deeply. In addition, in order to further improve the performance of the model in feature extraction and context awareness, we added residual connections between SCConv modules. These residual connections effectively realize the feature fusion between different SCConv modules, and ensure that the retention and utilization of feature information in the transmission process are more efficient. In this way, the network can better integrate and utilize multi-level feature information, thus improving the overall performance (Fig 5).

**Fig 6:**
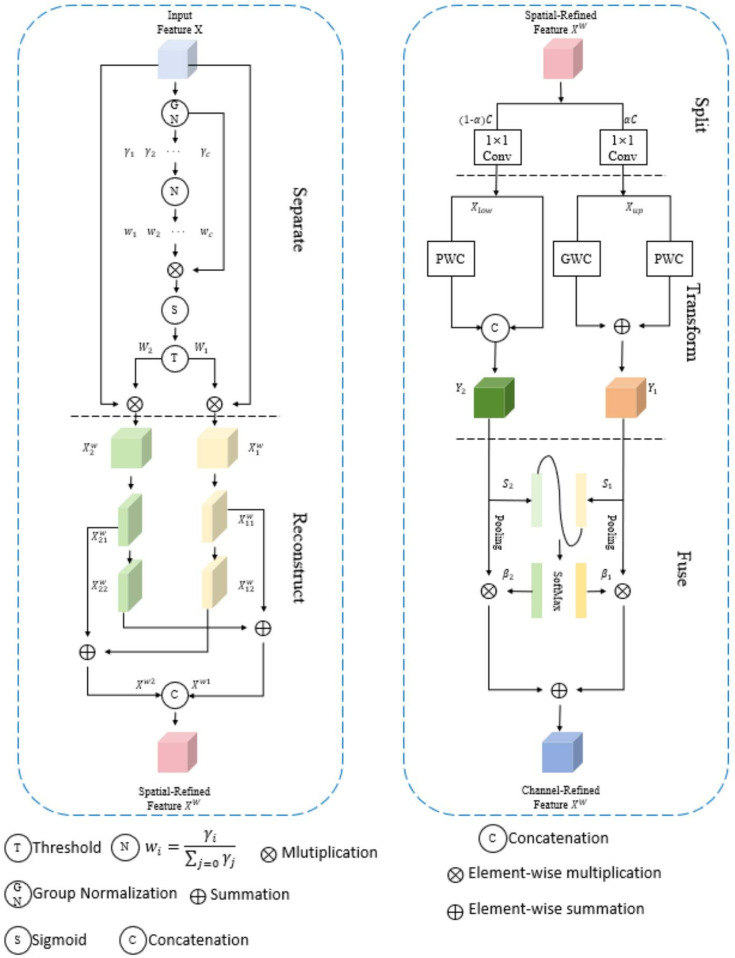
(a) SRU structure diagram; (b) CRU structure diagram.

The essence of the CRSCC module lies in its SCConv structure, as shown in Fig 6. This structure consists of two key components: the Spatial Reconstruction Unit (SRU) and the Channel Reconstruction Unit (CRU), whose detailed structures are shown in Fig 6, respectively. During the processing, the input feature X is first optimized through SRU, which improves the efficiency of spatial information processing through the following two main steps.

The first step is the spatial separation operation, which uses the scaling factor γ in group normalization to evaluate the information content of different feature mAPs, and then separates the feature mAPs with large information and the feature mAPs with small information into $X^{w1}$ and $X^{w2}$ parts. The second step is the spatial reconstruction operation. Through cross reconstruction, the weighted two different information features are combined to generate features with richer information and optimize the space occupation. The reconstruction operation is shown in Equation (1):


$$\left\{ \begin{array}{c} X_{1}^{w}=W_{1}⨂X,X_{2}^{w}=W_{2}⨂X\\ X_{11}^{w}{⨁X}_{22}^{w}=X^{w1},X_{21}^{w}{⨁X}_{12}^{w}=X^{w2}\\ X^{w1}\cup X^{w2}=X^{w} \end{array}\right.\;$$ (1)

Next, the spatially reconstructed feature $X^{w}$ enters the CRU to optimize the channel information. CRU contains three steps: segmentation, transformation, and fusion. In the segmentation stage, the feature $X^{w}$ is divided into two parts $X_{up}$ and $X_{low}$ with different channel numbers, where αC and (1−α)C represent their channel numbers respectively, and α is an adjustable hyperparameter. Subsequently, these two-part features are channel-compressed by a 1 × 1 convolution kernel.

In the conversion stage, $X_{up}$ is the main input of “rich feature extraction”, and the output $Y_{1}$ is obtained through the combination operation of packet convolution (GWC) and point convolution (PWC), as shown in equation (2). At the same time, $X_{low}$ as supplementary information is only processed by PWC, and its output is combined with the original $X_{low}$ to obtain $Y_{2}$, as shown in Equation (3).


$$Y_{1}=M^{G}X_{up}+M^{p1}X_{up}$$ (2)

$$Y_{2}=M^{p2}X_{low}\cup X_{low}$$ (3)

Finally, in the fusion stage, the global average pooling technique is used to obtain S1 and S2, and the feature weight vectors β1 and β2 are obtained by the Softmax function. These two weight vectors are used to combine Y1 and Y2 to generate the final channel purification feature Y. In this way, the CRSCC module can not only reduce redundant information, but also improve the quality of features, which is of great significance for small target detection.

#### Fusion multi-scale local feature coordinate attention.

PCB defect detection is an important link in electronic manufacturing industry, which is related to the quality and reliability of electronic products. With the development of miniaturization and high density of electronic products, the defect detection of PCB has become more and more challenging. The diversity of defect types, such as short circuit, open circuit, hole, foreign matter, offset, uneven copper foil thickness, each defect has different characteristics and detection difficulty. The interference of complex background, complex circuits on PCB and many background noises bring difficulties to defect identification. With the improvement of technology, the size of some defects is very small, which puts forward higher requirements for the resolution and sensitivity of the detection system. The balance between detection speed and accuracy requires fast detection speed on the production line, but at the same time, high accuracy must be ensured, which is a big challenge for detection algorithms and hardware equipment. Illumination and imaging problems, different lighting conditions may affect the quality of images, thus affecting the accuracy of defect detection.

In order to solve the above problems, in recent years, deep learning technology, especially attention mechanism, has been introduced into PCB defect detection. Attention mechanism can make the model pay more attention to the key areas related to defects in the image, while ignoring the unimportant background information, thus improving the detection accuracy. It can adaptively learn the feature representation of defects, especially in complex circuit background. In addition, the attention mechanism helps the model to focus on small changes in the image, which is particularly effective for the detection of small defects. By strengthening the model’s attention to defect areas, false detection caused by background interference can be reduced, and at the same time, the detection ability of small defects can be improved, and missed detection can be reduced. The attention mechanism also helps the network make more efficient use of computing resources, and reduces unnecessary calculations by focusing on key areas, thereby improving detection speed.

**Fig 7:**
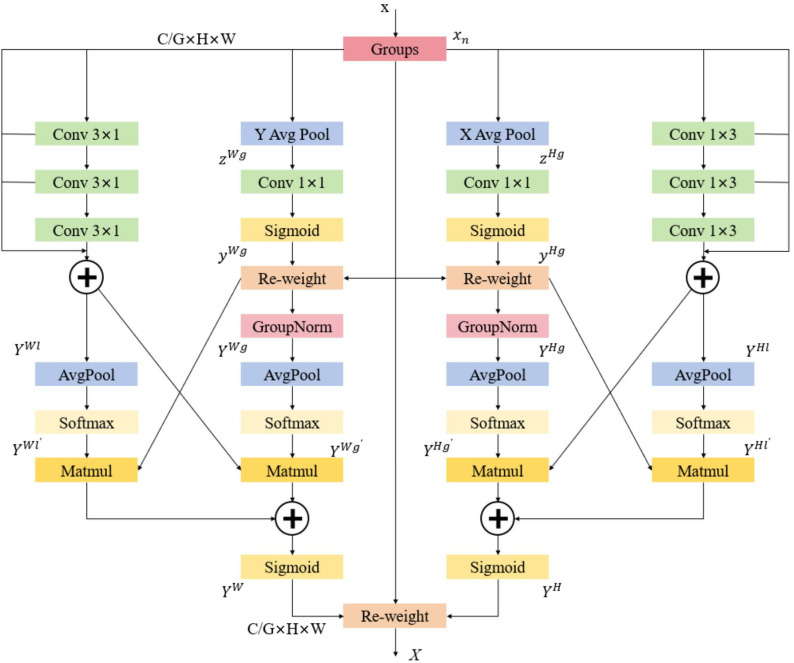
FFCA attention module structure.

**Fig 8:**
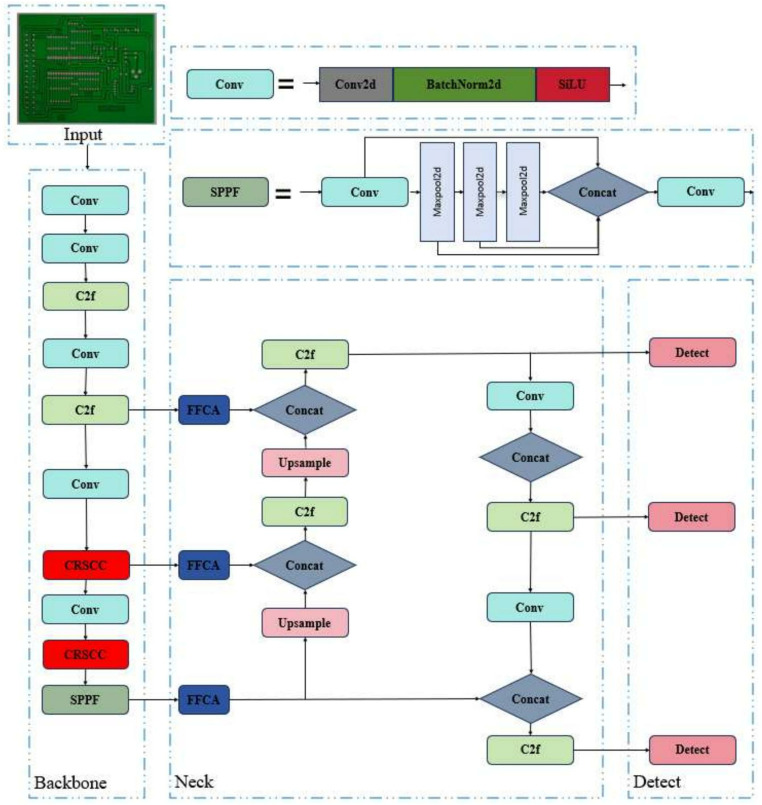
PCB-YOLO model structure.

The coordinate attention (CA) [30] module pools in two different directions, aggregates the input feature mAP information horizontally and vertically, and embeds the position information into the channel attention, realizing the interaction of two dimensions. However, the CA module only pays attention to the global position information and long-range dependency of the corresponding direction, but does not pay attention to the local position information. In order to solve the above problems, this paper designs the FFCA attention mechanism, and its structure is shown in Fig 7. FFCA mainly includes three parts: feature grouping, multi-branch features and spatial information aggregation (Fig 8).

The dimension of the input feature map of the FFCA module is C×H×W, which represents the number of channels, height and width of the feature map respectively. The feature map is divided into G sub-features according to the number of channels, and each group is isolated from each other, and subsequent operations are performed separately to learn different features. G is generally much smaller than C to ensure the generalization ability of each group.

Multi-branch features use different methods to process the input feature map, from left to right, they are vertical local features, vertical global features, horizontal global features and horizontal local features respectively. The input feature $x_{n}$ is denoted as x for convenience of representation. In the horizontal global feature branch, the grouped input feature maps aggregate features along the horizontal direction to obtain long-range information and retain accurate information in the vertical direction. Specifically, the input information is pooled using a pooling kernel of size (W,1to generate a feature map $z^{Hg}$ of size C/G×H×1, and H,W,g,l in the upper corner indicates horizontal, vertical, global, and local respectively. The pooled output is expressed as Equation (4), where W represents the width of the feature map and h represents the h throw of the feature map.


$$z^{Hg}(h)=\frac{1}{W}\sum_{0\leq i<W}x(h,i)$$ (4)

Next, keep the number of channels unchanged, use convolution to enhance the feature information, and obtain the final weight $y^{Hg}$ through the Sigmoid activation function, as shown in Equation (5), where σ represents the Sigmoid activation function, $F_{1\times 1}$ represents convolution 1 * 1, and $z^{Hg}$ represents the pooled sequence feature. After that, the input features are adjusted according to the weights to obtain the horizontal global feature $Y^{Hg}$, which is expressed as Equation (6), and $G_{n}$ represents group normalization:


$$y^{Hg}=\sigma \left(F_{1\times 1}\left(z^{Hg}\right)\right)$$ (5)

$$Y^{Hg}=G_{n}\left(\left(x\times y^{Hg}\right)\right)$$ (6)

Similarly, the pooled features in the vertical global feature branch and the vertical global features are expressed as equations (7), (8), where H represents the height of the feature mAP and w represents the w-th row of the feature mAP.


$$z^{Wg}(h)=\frac{1}{H}\sum_{0\leq i<H}x(i,w)$$ (7)

$$Y^{Wg}=G_{n}\left(x\times \sigma \left(F_{1\times 1}\left(z^{Wg}\right)\right)\right)$$ (8)

In the horizontal local feature branch, multiple 1 × 3 convolutions are used to simulate the effects of 1 × 3, 1 × 5, 1 × 7 convolutions, and fewer parameters are used to simulate multi-scale feature information to obtain the horizontal local feature $Y^{HI}$. Equation is shown in (9), where $F_{1\times 3}^{n}$ represents n times of 1 × 3 convolutions. Similarly, the vertical local feature is expressed as equation (10), where $F_{3\times 1}^{n}$ represents n times of 3 × 1 convolution, l represents local, and $Y^{HI}$ is a horizontal local feature. The characteristics of the four branches are denoted by $Y^{Hg}$, $Y^{Wg}$, $Y^{HI}$, $Y^{WI}$ respectively.


$$Y^{Hl}=F_{1\times 3}^{3}(x)+F_{1\times 3}^{2}(x)+F_{1\times 3}^{1}(x)+x$$ (9)

$$\;Y^{Wl}=F_{1\times 3}^{3}(x)+F_{1\times 3}^{2}(x)+F_{1\times 3}^{1}(x)+x\;$$ (10)

Spatial information aggregation: After multi-branch processing, global and local information in two directions are obtained, which are relatively independent. In order to achieve more comprehensive information description, the spatial information aggregation module is used to promote the interaction between them. In the horizontal branch, the outputs $Y^{Hg}$, $Y^{Hl}$ of the two branches are globally coded as $Y^{Hg^{\prime }}$, $Y^{Hl^{\prime }}$ by global average pooling.


$$Y^{Hg^{\prime }}=S\left(\frac{1}{W\times H}\sum_{0\leq j<H}\sum_{0\leq I<W}Y^{Hg}(j,i)\right)$$ (11)

$$Y^{Hl^{\prime }}=S\left(\frac{1}{W\times H}\sum_{0\leq j<H}\sum_{0\leq I<W}Y^{Hl}(j,i)\right)$$ (12)

The global spatial features of the two branches are multiplied by the matrix dot product operation to generate the first spatial attention mAP. Then, a second spatial attention mAP is generated in the same way, which retains the complete precise spatial location information. The weights in the horizontal direction are added by the two generated spatial attention mAPs, and then the input is compressed using the Sigmoid function to obtain the horizontal spatial weight $Y^{H}$, as shown in Equation (13). In the same way, the vertical space weight $Y^{W}$ is obtained.


$$Y^{H}=\sigma \left(Y^{Hg^{\prime }}Y^{Hl}+Y^{Hl^{\prime }}Y^{Hg}\right)$$ (13)

Finally, the initial feature x is multiplied by the two dimensional weights to obtain the FFCA attention feature X, as shown in Equation (14).


$$X=Y^{H}Y^{W}x$$ (14)

#### Loss function improvement.

The calculation of loss function is an important part of object detection algorithm, and the prediction box regression loss function of YOLOv8s algorithm defaults to CIoU [31]. CIoU is the calculation of the loss of the bounding box, mainly considering three important geometric factors: the overlapping area of the predicted box and the real box, the distance from the center point, and the predicted box and aspect ratio. The specific formula of CIoU is as follows:


$$CIoU=IoU-\left(\frac{\rho^{2}\left(B^{pred},B^{gt}\right)}{c^{2}}+\alpha v\right)$$ (15)

$$v=\frac{4}{\pi }\left(\arctan\frac{w^{gt}}{h^{gt}}-arctan\frac{w^{pred}}{h^{pred}}\right)$$ (16)

$$\alpha =\frac{v}{(1-IoU)+v}$$ (17)

Among them, α is used as a balance parameter, v is used to measure the length and width, and $\rho^{2}\left(B^{pred},B^{gt}\right)$ represents the Euclidean distance between the prediction box and the center point of the real box. However, CIoU does not consider the quality of the labeled examples of the data set itself and ignores the impact of low-quality data on detection performance. harm. Therefore, this paper first introduces Wise-IoU [32] as a new bounding box loss function.

βis used to denote the anomaly degree of the prediction box, which is defined as follows:


$$\beta =\frac{L_{IoU}^{*}}{L_{IoU}}\in [0,+\infty )\;$$ (18)

Where $L_{IoU}^{*}$ is the constant converted from the variable $\overset{―}{L_{IoU}}$, $\overset{―}{L_{IoU}}$ is the running average of the momentum m, and the calculation formula of m is:


$$m=\sqrt[{tn}]{0.05}$$ (19)

Where t is the Epoch value; n is the value of Batch Size.

Wise-IoU is defined as:


$$L_{WIoU}=\frac{\beta }{\delta \alpha_{1}^{\beta -\delta }}R_{WIoU}L_{IoU}$$ (20)

$$R_{WIoU}=exp\left(\frac{{\left(x-x_{gt}\right)}^{2}+{\left(y-y_{gt}\right)}^{2}}{{\left(W_{g}^{2}+H_{g}^{2}\right)}^{*}}\right)$$ (21)

$$L_{IoU}=1-IoU$$ (22)

Among them, (x,y) and $\left(x_{gt},y_{gt}\right)$ are coordinates of the center points of the prediction frame and the target frame respectively; $W_{g}$ and $H_{g}$ are the dimensions of the smallest bounding box; IoU is the cross-to-merge ratio, which is a commonly used quantity to measure the degree of overlap between the predicted frame and the real frame. $\alpha_{1}$ and δ are learning parameters. Wise-IoU reduces the detrimental gradients of low-quality data, thereby improving the overall performance of the model.

But Wise-IoU is not aware of the limitations of IoU itself. Inner-IoU [33] proposes to calculate IoU with an auxiliary border to improve the generalization ability. The specific calculation process is shown in equations (23) and (24), and the scale factor ratio is used to control the size of the auxiliary bounding box.


$$b_{l}=x_{c}-\frac{w\times ratio}{2},b_{r}=x_{c}-\frac{w\times ratio}{2}$$ (23)

$$b_{t}=y_{c}-\frac{h\times ratio}{2},b_{b}=y_{c}-\frac{h\times ratio}{2}$$ (24)

Through the above two equations, the center point of the detection frame can be transformed to obtain the corner vertices of the auxiliary detection frame. Both the prediction box and the real box output by the model are transformed accordingly, and the calculation results of the real box and the prediction box are represented by $b^{gt}$ and $b^{pred}$ respectively.


$$\;S_{inter}=\left(\min\left(b_{r}^{gt},b_{r}\right)-max\left(b_{l}^{gt},b_{l}\right)\right)\times \left(\min\left(b_{b}^{gt},b_{b}\right)-max\left(b_{t}^{gt},b_{t}\right)\right)$$ (25)

$$S_{union}=\left(w^{gt}\times h^{gt}\right)\times (ratio{)}^{2}+(w\times h)(ratio{)}^{2}-S_{inter}$$ (26)

$${IoU}^{inner}=\frac{S_{inter}}{S_{union}}$$ (27)

According to the above three formulas, it can be seen that Inner-IoU actually calculates the IoU between the auxiliary borders. When ratio = 1, the Inner-IoU loss function is actually the IoU loss function. Since the PCB defect detection task images are all small targets, the IoU will decrease if the labeling box is slightly offset. When ratio > 1, the auxiliary border is larger than the actual border, which is helpful for sample regression with low IoU. Therefore, for small target detection, the value of ratio should be greater than 1.

MPDIoU is an improved regression loss function that can minimize the distance between the upper left corner and the lower right corner of the prediction box and the real box [34]. It can well deal with the overlap of bounding boxes, has a good effect on complex multi-objective scenes, and can improve the convergence speed.


$$MPDIoU=IoU-\frac{\rho^{2}\left(P_{1}^{pred},P_{1}^{gt}\right)}{w^{2}+h^{2}}-\frac{\rho^{2}\left(P_{2}^{pred},P_{2}^{gt}\right)}{w^{2}+h^{2}}$$ (28)

Among them, $P_{1}^{pred}$, $P_{2}^{pred}$, $P_{1}^{gt}$ and $P_{2}^{gt}$ refer to the points in the upper left corner and the lower right corner of the prediction box and the real box respectively, and $\rho^{2}\left(P_{1}^{pred},P_{1}^{gt}\right)$ calculates the distance between the corresponding points.

Combining inner-IoU and MPDIoU, Inner-MPDIoU is obtained, as shown in equation (29), which can not only calculate IoU through the auxiliary border, improve the generalization ability, but also solve the overlapping problem of bounding boxes in complex multi-objective scenes.


$${MPDIoU}^{inner}={IoU}^{inner}-\frac{\rho^{2}\left(P_{1}^{pred},P_{1}^{gt}\right)}{w^{2}+h^{2}}-\frac{\rho^{2}\left(P_{2}^{pred},P_{2}^{gt}\right)}{w^{2}+h^{2}}$$ (29)

On the basis of obtaining inner-MPDIoU, combining Wise-IoU can reduce the harm of low-quality data to detection performance. At the same time, replacing the IoU calculation part of Wise-IoU with the obtained inner-MPDIoU, IoU can be calculated through the auxiliary border, solving the limitation of IoU itself, improving the generalization ability of the model, and finally obtaining the improved loss function WIPIoU, that is, equation (30), which can effectively improve the model detection effect.


$$L_{WIPIoU}=\frac{\beta }{\delta \alpha_{1}^{\beta -\delta }}\times exp\left(\frac{{\left(x-x_{gt}\right)}^{2}+{\left(y-y_{gt}\right)}^{2}}{{\left(W_{g}^{2}+H_{g}^{2}\right)}^{*}}\right)\times \left(1-{IoU}^{inner}+\frac{\rho^{2}\left(P_{1}^{pred},P_{1}^{gt}\right)}{w^{2}+h^{2}}+\frac{\rho^{2}\left(P_{2}^{pred},P_{2}^{gt}\right)}{w^{2}+h^{2}}\right)$$ (30)

#### Overall network architecture.

**Fig 9:**
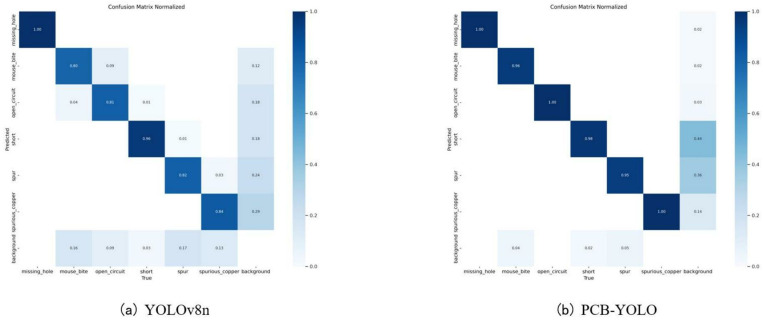
Confusion matrix.

As shown in Fig 9, the PCB-YOLO model consists of three parts: the backbone network, the neck network and the head. The trunk part is responsible for extracting feature information and generating multi-scale features. A novel CRSCC module based on SCConv convolution and C2f is proposed, which significantly reduces the number of parameters of the model through spatial and channel feature purification, while improving the accuracy of PCB defect detection, effectively solving the problem of bulky model volume; According to the characteristics of PCB surface defects, the FFCA attention module is designed. By fusing multi-scale local features, it can capture remote spatial dependence and multi-scale accurate local position information, enhance detail attention, and improve the resolution ability and detection effect of features; The WIPIoU loss function is designed, and the IoU is calculated by auxiliary boundary, and the influence of low-quality data is considered, which improves the recognition accuracy of the model for small targets and accelerates the convergence speed of the model.

## Results and analysis

### Experiment environment

The operating system version used in this experiment is Ubuntu 18.04.5 LTS, the CPU is AMD Ryzen 5900HX processor, the GPU is NVIDIA GeForce RTX 3090 (24GB), the CUDA version is 11.7, the deep learning framework is Pytorch 1.13.1, and the compilation environment is Python 3.9. Model The detailed parameters for training are shown in Table 3.

**Table 3:** Model training parameters.

Hyperparameters	Value
Initial learning rate	0.01
Optimizer	SGD
Optimizer momentum	0.937
Optimizer weight decay rate	0.0005
Batchsize	16
Epoch	300

### Evaluation metrics

A total of five evaluation metrics are employed in order to provide a comprehensive assessment of the detection model. These include precision, recall, parameter count, computational complexity, and mean average precision (mAP). The equations for these metrics are as follows:


$$P=\frac{TP}{TP+FP}$$ (31)

$$R=\frac{TP}{TP+FN}$$ (32)

$$P=\int_{0}^{1}P(R)dR$$ (33)

$$mAP=\frac{\sum_{0}^{1}{AP}_{1}}{n}$$ (34)

TP represents the number of positive samples correctly predicted as positive by the model, FP represents the number of negative samples incorrectly predicted as positive by the model, and FN represents the number of positive samples incorrectly predicted as negative by the model. AP represents the area under the Precision-Recall (P-R) curve, while mAP denotes the average of AP for each category.

### Analysis of the impact of CRSCC modules in different positions on model performance

In order to determine where the CRSCC module is added in the backbone network of YOLOv8n model to have the best comprehensive improvement effect on each index, experiments are carried out for the following four situations. Although the improved CRSCC module has stronger feature extraction ability and lower parameter quantity in theory, because the improvement is carried out for C2f in the deep backbone network, there is uncertainty whether any improvement of C2f can achieve performance improvement. Therefore, a set of comparative experiments are carried out to improve CRSCC modules in different locations. Backbone 3_5_7_9 means to improve the C2f modules of layers 3, 5, 7 and 9 in the backbone network, Backbone 5_7_9 means to improve the C2f modules of layers 5, 7 and 9 in the backbone network, Backbone 7_9 means to improve the C2f modules of layers 7 and 9 in the backbone network, and Backbone9 means to improve only the deepest C2f modules in the backbone network. The experimental results are shown in Table 4. The experimental results show that although CRSCC module has lower parameters and stronger ability to extract features than C2f in theory, the more improved modules, the better. The model has the best performance when the improvement module acts on the deeper network.

**Table 4:** Comparison of CRSCC module improvement in different locations.

Model	Parameters(MB)	Precision/%	Recall/%	mAP50/%
YOLOv8n	3.0	**94.5**	88.9	95.7
Backbone3_5_7_9	2.2	93.6	89.3	94.9
Backbone5_7_9	2.3	93.7	89.9	96.1
Backbone7_9	2.6	93.3	**91.4**	**96.3**
Backbone9	2.9	94.0	88.7	95.6

#### Attention mechanism contrast test.

In order to verify the superiority of the FFCA attention mechanism proposed in this paper, we use very popular attention modules for comparative experiments, such as SE [35], GAM [36], SimAM [37], CBAM [38], ECA [39], EMA [40] and CA.

The experimental results are shown in Table 5. It can be seen from Table 5 that most attention modules have a positive effect on the model after being added, and only SE attention module and GAM attention module will have a negative impact on the model. Adding FFCA module to the model improves the detection effect greatly, which proves the effectiveness and practicability of FFCA attention module.

**Table 5:** Analysis of influence of different attention modules on model performance.

Model	Precision/%	Recall/%	mAP50/%
YOLOv8n	94.5	88.9	95.7
YOLOv8n+SE	92.7	89.2	94.3
YOLOv8n+GAM	94.1	86.9	95.3
YOLOv8n+SimAM	94.7	87.8	96.7
YOLOv8n+CBAM	95.0	87.1	97.1
YOLOv8n+ECA	94.5	88.4	96.3
YOLOv8n+EMA	93.0	87.6	97.4
YOLOv8n+CA	94.8	88.5	97.7
YOLOv8n+FFCA	95.3	89.1	98.4

#### Analysis of the influence of WIPIoU loss function on model performance.

In this chapter, we conduct quantitative comparison experiments on several loss functions: CIoU, GIoU [41], SIoU [42], ShapeIoU [43], Inner-IoU, DIoU and WIPIoU. The experimental results are shown in Table 6. By comparing the experimental results, we find that the model accuracy, recall rate and mAP50 using the WIPIoU loss function are better than other loss functions. The accuracy of the model using WIPIoU loss function is significantly improved, which shows that the model reduces misjudgment when identifying defects and improves the detection accuracy. The performance of the WIPIoU loss function in recall rate is also excellent, indicating that the model can effectively identify more real defects and reduce missed detection, which is particularly important for PCB surface defect detection in practical applications.

**Table 6:** Analysis of the impact of loss function on model performance.

Model	Precision/%	Recall/%	mAP50/%
YOLOv8n	94.5	88.9	95.7
YOLOv8n+Inner-IoU	94.3	88.2	95.8
YOLOv8n+GIoU	94.8	89.0	95.5
YOLOv8n+SIoU	94.7	88.2	95.5
YOLOv8n+Shape-IoU	94.9	88.8	96.0
YOLOv8n+DIoU	93.3	89.1	94.9
YOLOv8n+WIPIoU	**95.1**	**89.3**	**96.2**

#### PCB-YOLO model performance analysis.

We further evaluate the detection ability of the trained model through the confusion matrix. The confusion matrix is shown in Fig 9. From the confusion matrix, it can be seen that the improved model can reflect higher classification accuracy in the identification of various defects on the PCB surface, and the classification performance is more balanced. The background false judgment rate of PCB surface defects is high, which shows that the complex environment will interfere with the recognition of detection targets by the model, and there is still the problem of missed detection of PCB surface defects, which may be due to the complexity of the environment that makes the characteristics of tiny defects difficult to identify and capture.

**Fig 10:**
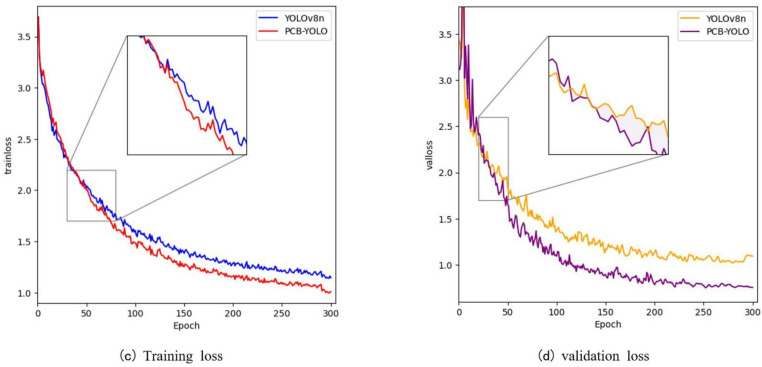
Training loss and validation loss.

The relationship between training loss and verification loss is an important indicator to measure model performance. In order to clearly describe the changing trend of loss function during model iteration, we draw a comparison of training loss and verification loss curves of YOLOv8n and PCB-YOLO models, as shown in Fig 10.

From the analysis of Fig 10, it can be seen that the training loss and verification loss of our proposed PCB-YOLO model both decrease slowly at the same time and tend to a specific threshold, with little difference, and both of them are lower than the YOLOv8n model, which shows that the WIPIoU algorithm can more accurately transmit the error back along the gradient minimum path direction, and update the weight value to speed up the convergence speed and improve the detection accuracy.

#### Ablation experiment.

In order to intuitively demonstrate the performance of the improved method in PCB defect detection, a series of ablation experiments are designed with YOLOv8n model as the benchmark model, which is verified by one-by-one ablation, in which “√” indicates joining the module, the experimental results are shown in Table 7, and the bold part indicates the optimal value.

**Table 7:** Ablation experiment.

CRSCC	FFCA	WIPIoU	Parameters(MB)	FLOPs (G)	Precision/%	Recall/%	mAP50/%
			3.0	8.1	94.5	88.9	95.7
√			2.6	6.9	94.9	88.4	96.3(+0.6)
√	√		2.6	6.9	95.3	89.1	98.4(+2.7)
√	√	√	2.6	6.9	95.2	89.7	98.8(+3.1)

It can be seen from Table 7 that after first replacing the C2f module in the backbone network with CRSCC module, the mAP50 value increases by 2.5%, indicating that CRSCC module can effectively improve the model feature fusion ability and the global feature information extraction ability of defect feature image; Then, after adding the FFCA attention module to the feature fusion network part, mAP50 increased by 2.2%, indicating that our proposed FFCA module enables the network to better utilize low-level and high-level features, improve the accuracy and robustness of detection, and improve the detection effect of small targets; Finally, the CIoU loss function is replaced by the WIPIoU loss function, and the mAP value is increased by 0.4%, which shows that the improved loss function can effectively improve the robustness of the model. The results of ablation experiments show that the improvements made to YOLOv8n model in this paper can improve its detection accuracy, which shows the rationality and effectiveness of PCB-YOLO algorithm. To sum up, the various improved methods proposed in this paper have positive effects on PCB surface defect detection.

#### Comparative analysis with other models.

In order to verify the performance of the improved model in PCB surface defect detection tasks more deeply, the improved model is compared with the mainstream target detection models, including Faster-RCNN [44], SSD [45], Rtdetr-l [46], YOLOv7-tiny, YOLOv8n, YOLOv10n [47], and YOLO11n models, and other state-of-the-art methods such as YOLOv5-SSCD [48], Improved-YOLOv5s [49], CDS-YOLO [50], CGS-YOLOv5 [51], YOLO-HMC [52], YOLO-World [53], Mask DINO [54], EffNet-YOLO [55], YOLOv8-PCB [56], MS-DETR [57]. Table 8 gives a comparative analysis of our proposed method and the above method on a series of indicators, including parameter quantity, calculation quantity, accuracy, recall rate and average accuracy mean, where bold indicates the optimal value.

**Table 8:** PCB-YOLO compared with other different models.

	Model	FLOPs(G)	Parameters(MB)	Precision/%	Recall/%	mAP50/%	FPS
Advanced two-stage methods	Faster R-CNN	136.8	/	93.3	81.1	88.3	17.4
Advanced one-stage methods	SSD	12.3	38.8	92.0	89.7	93.4	22.1
	Rtdetr-l	32.0	103.5	95.2	97.1	97.3	19.7
	YOLOv7-tiny	13.9	6.2	92.9	94.1	94.6	62.5
	YOLOv8n	3.0	8.1	94.5	88.9	95.7	94.3
	YOLOv10n	2.6	8.2	93.0	94.9	95.1	73.6
	YOLO11n	**2.5**	6.3	91.6	95.2	94.4	75.5
Current SOTA methods	YOLOv5-SSCD	25.4	/	96.3	89.1	94.5	/
	Improved-YOLOv5s	16.4	46.6	/	97.2	96.1	/
	CDS-YOLO	13.5	/	**97.4**	92.9	95.3	/
	YOLO-World	13.38	38.4	84.7	80.4	83.6	44.1
	Mask DINO	223	1326	82.1	77.3	81.0	9.2
	CGS-YOLOv5	/	8.2	/	/	95.4	/
	YOLO-HMC	/	5.94	/	/	98.6	/
	EffNet-PCB	22.2	12.6	96.2	96.1	95.6	/
	YOLOv8-PCB	7.1	**5.2**	94.7	94.0	96.1	/
	MS-DETR	45.6	14.0	/	/	96.9	**115**
Proposed	PCD-YOLO	2.6	6.9	96.3	**97.9**	**98.8**	102.8

It can be seen from Table 8 that the Faster-RCNN model is the largest and has the largest number of parameters, but the average accuracy is the lowest, and the detection effect is poor; The Rtdeter-l model is to improve the model with the best detection effect, but it has the highest amount of calculation and cannot exert full performance on devices with low computing power; The parameters of YOLOv5n model and YOLO11n model are the smallest, both are 2.5, but the detection results are average; Comparing PCB-YOLO with the current SOTA method, YOLOv5-SSCD, Improved-YOLOv5s, CDS-YOLO, and CGS-YOLOv5 models have larger sizes but lower accuracy; YOLO-World and Mask DINO have the lowest detection accuracy when there are many parameters, while CGS-YOLOv5 has lower detection accuracy; YOLO-HMC has high detection accuracy with good model size control; The EffNet-PCB model has a larger size and slightly lower accuracy; The YOLOv8-PCB model has the fewest parameters, but slightly lower detection accuracy; Although PCB-YOLO has a lower detection speed than MS-DETR, MS-DETR has a larger number of parameters and calculations, and its detection accuracy is not high; The PCB-YOLO model proposed in this paper shows the strongest performance, with an average accuracy of 98.8, which is 3.1 percentage points higher than the benchmark model. In terms of parameters, it meets the requirements of lightweight, and at the same time, it can be easily run on equipment with low computing power. The experimental results clearly show that our method is superior to other methods in overall performance.

#### Visualization experiments.

**Fig 11:**
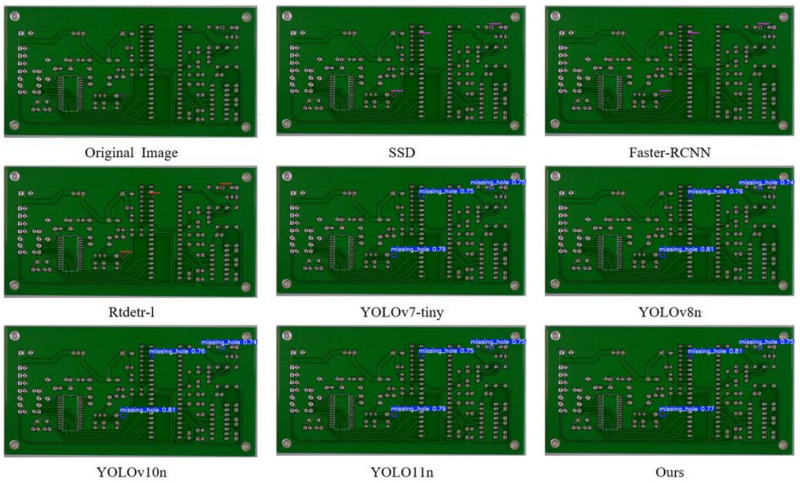
Missing hole S defect detection diagram.

**Fig 12:**
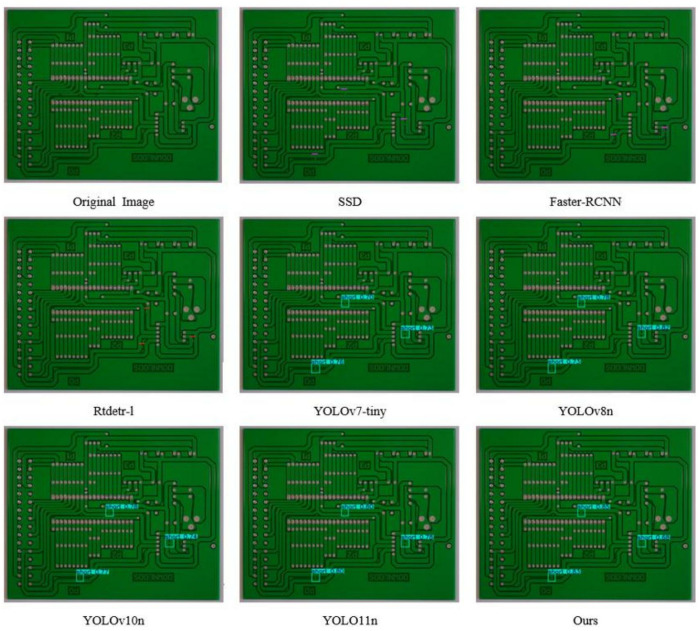
Short defect detection diagram.

**Fig 13:**
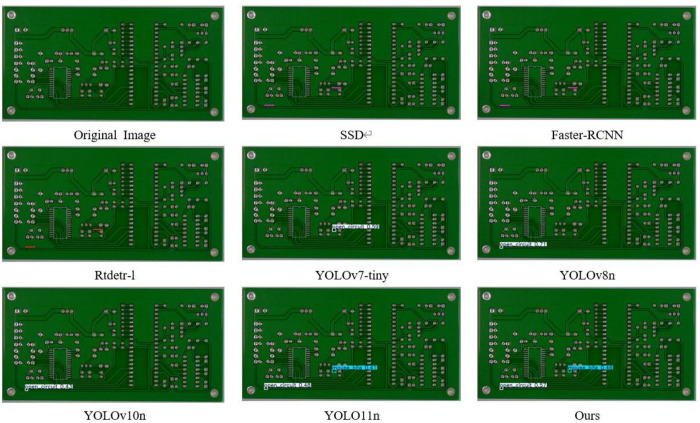
Open circuit defect detection diagram.

**Fig 14:**
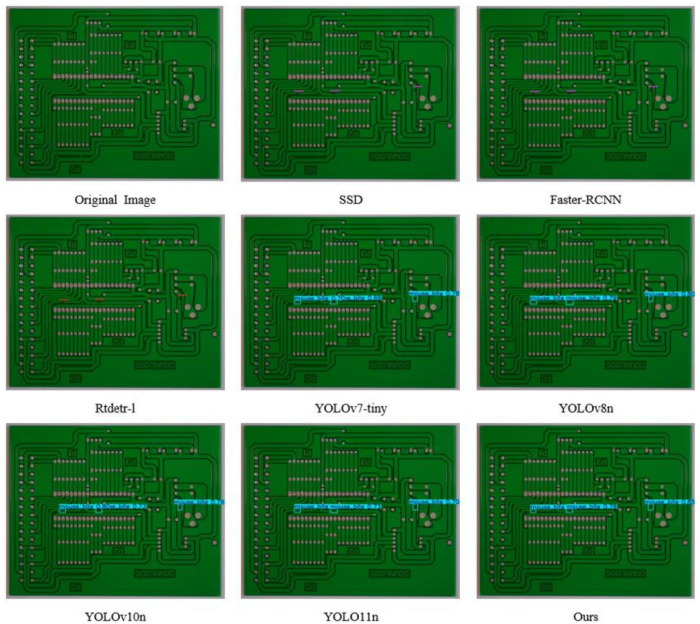
Mouse bite defect detection diagram.

**Fig 15:**
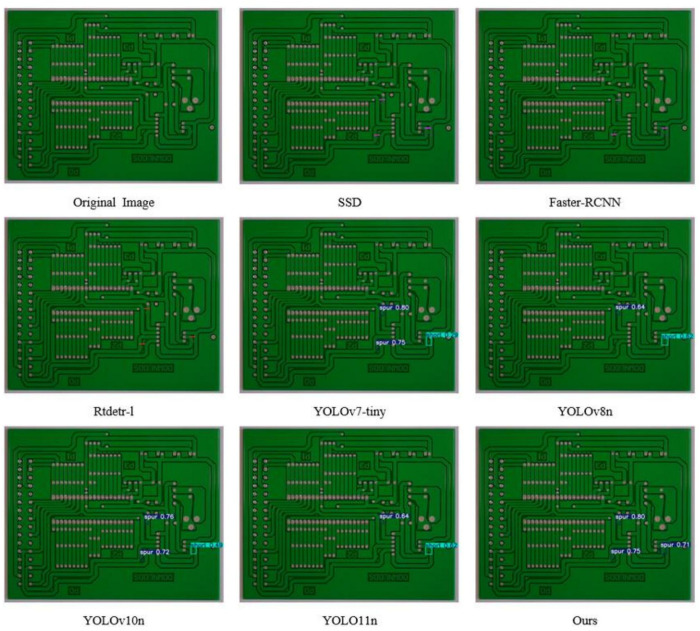
Spur defect detection diagram.

**Fig 16:**
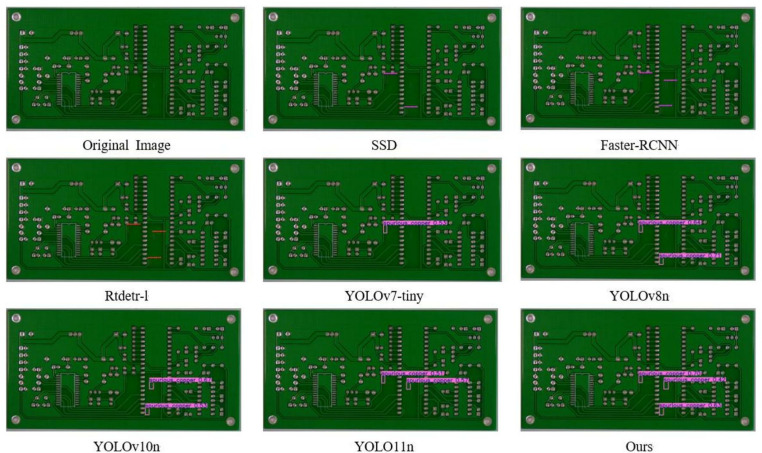
Spurious copper defect detection diagram.

In order to visually demonstrate the advantages of this algorithm in detection performance, we use the PCB-YOLO model and several other different detection algorithms to conduct comparative experiments on PCB surface defect datasets, and different types of defect types are labeled by different color borders. The experimental results are shown in Figs 11–16. It can be seen from the figure that in some defect detection with obvious features, all algorithms can detect defects, but when the defect features are not obvious, most models have serious false detection and missed detection. In contrast, our algorithm performs better at identifying PCB surface defects. We can see that this algorithm has made significant progress in the detection of spur and spurious copper defects. This is also clearly reflected in the visualization results. Especially in the aspect of difficult detection defects, other algorithms generally have a high missed detection rate, but our algorithm can accurately mark multiple defects, which shows that the optimized algorithm has stronger ability in shallow feature extraction and fusion, and effectively reduces the missed detection phenomenon, especially in the detection of small target defects.

#### Model generalization verification.

In order to further verify the effectiveness and generality of the algorithm, in addition to validating it on the PCB surface defect dataset, the NEU-DET steel surface defect dataset provided by Northeastern University was also used for validation. This dataset contains a total of 1800 images with a size of 200 × 200 pixels, covering six types of defects: silver lines, inclusions, patches, rough surfaces, scratches, and dents. Each type of defect has 300 images. The dataset is divided into a training set, a validation set, and a testing set in a ratio of 6:2:2, with 1080 images in the training set, 360 images in the validation set, and 360 images in the testing set. Compared PCB-YOLO, YOLOv8n, MD-YOLO [58], Mask-DINO, YOLO -World, DF-YOLOv7 [59], BiCCFM [60],GLF-NET [61].

The experimental results are shown in Table 9. Compared with YOLOv8n, the average accuracy of PCB-YOLO is improved by 5.7%, the amount of parameters is reduced by 13.3%, and the amount of calculation is reduced by 14.8%; Compared with the improved version of YOLOv5, the average accuracy of MD-YOLO is increased by 1 percentage point; Compared to Mask DINO, PCB-YOLO significantly improved the mean average precision by 17.4%, while reducing the number of parameters by 98.8% and computational load by 99.5%. Compared to YOLO-World, PCB-YOLO improved the mean average precision by 12.9%, reduced the number of parameters by 80.6%, and decreased the computational load by 82.0%. Compared with the DF-YOLOv7 model, the average accuracy of PCB-YOLO has been improved by 2.1 percentage points, and the computational load has been slightly reduced by 4.5 G FLOP; Compared with BiCCFM, PCB-YOLO improved the average accuracy by 5 percentage points using approximately the same number of parameters and less than half of the FLOP calculation; GLF-NET achieves the same average accuracy as our proposed PCB-YOLO, but the computational complexity and model complexity are relatively high. The experimental results demonstrate that the improved model has enhanced accuracy, proving the greater potential of the proposed model in industrial applications. Furthermore, the algorithm exhibits significant advantages in terms of parameter count and computational efficiency, further validating its strong generalization performance across different datasets.

**Table 9:** Comparative experiment of model generalization.

Model	Parameters(MB)	FLOPs (G)	Precision/%	Recall/%	mAP50/%
YOLOv8n	3.0	8.1	73.3	67.9	73.7
MD-YOLO	9.0	14.1	72.5	**72.9**	78.2
Mask DINO	223	1326	64.3	59.8	61.8
YOLO-World	13.38	38.4	70.1	64.4	66.3
DF-YOLOv7	/	12.4	/	/	77.1
BiCCFM	12.2	33.2	/	/	74.2
GLF-NET	12.0	34.6	/	/	**79.2**
PCB-YOLO	**2.6**	**6.9**	**78.3**	70.7	**79.2**

## Conclusions

In this paper, a PCB-YOLO model based on YOLOv8 framework is proposed to solve the problems of low detection accuracy and low detection efficiency in PCB surface defect detection. First of all, aiming at the problem that the existing PCB surface defect detection model has a large number of parameters and is not easy to deploy on edge devices, we propose a CRSCC module to replace the C2f module of the feature extraction part. Through feature purification in space and channels, the redundant information of the feature mAP50 improves the quality of the feature. The purified feature mAP50 contains richer information, which helps the model better identify the target, improve the detection ability of the model pair, and reduce the amount of model parameters and calculations; Then, aiming at the problem that there are many small target defects on the PCB surface, we propose a brand-new coordinate attention mechanism MLFCA that fuses multi-scale local features. This module can simultaneously capture remote spatial dependencies and multi-scale accurate local position information, enhance the attention to details, effectively solve the problem of position information loss in traditional global pooling, and improve the feature resolution ability and detection effect; Finally, we use the WIPIoU loss function instead of the CIoU function. WIPIoU calculates IoU through the auxiliary border and considers the influence of low-quality data, which effectively improves the model’s recognition accuracy of small targets. In addition, WIPIoU combines the advantages of Inner-IoU and MPDIoU. It improves the generalization ability of the model and accelerates the convergence speed of the model.

Experiments were conducted on the public data set PKU-Market-PCB. The experimental results show that the PCB-YOLO model has greatly improved the PCB surface defect task compared with the benchmark model. It is worth noting that mAP50 has improved by 5.7%, and The amount of model parameters and calculations were reduced by 13.3% and 14.8% respectively. The mAP50 value is 8.5%, 5.1%, 7.5% and 5.8% higher than that of YOLOv7-tiny, YOLOv8n, YOLOv10n and YOLO11n, respectively. Taken together, our model has the best overall performance. Despite this, the PCB-YOLO model still has room for improvement in detection accuracy. In the future, we will further study and explore other improvement methods, such as introducing more advanced attention mechanisms, data enhancement technologies, etc., to further improve the detection accuracy and robustness of the model.
